# *Chlamydia trachomatis* in the gingival sulcus and pharynx in patients of Northeast Mexico

**DOI:** 10.1002/cre2.290

**Published:** 2020-03-27

**Authors:** Erika E. Coronado‐Cerda, Jesus Ancer‐Rodríguez, Raul Montemayor‐Martínez, Fabrizio Canabal‐Hermida, Guadalupe Gallegos‐Avila, Myriam A. De la Garza‐Ramos

**Affiliations:** ^1^ Escuela Ciencias de la Salud, Universidad del Valle de México Monterrey Nuevo Leon Mexico; ^2^ Facultad de Odontología/Posgrado de Periodoncia y Unidad de Odontología Integral y Especialidades del CIDICS, Universidad Autónoma de Nuevo León Monterrey Nuevo León Mexico; ^3^ Departamento de Patología, Facultad de Medicina y Hospital Universitario Dr. Jose Eleuterio Gonzalez, Universidad Autónoma de Nuevo León Monterrey Nuevo Leon Mexico

**Keywords:** *Chlamydia trachomatis*, extragenital infection, oral health disease

## Abstract

**Background:**

The oral microenvironment provides the conditions for the establishment of microorganisms not usually considered residents of the normal oral microbiota. Sexually transmitted microorganisms such as *Chlamydia trachomatis* can adhere to any mucosal surface and ascend to reach appropriate locations to survive and develop symptomatic infections.

**Materials and methods:**

To determine the presence of *C. trachomatis*, direct immunofluorescence of this microorganism was carried out in 76 randomly selected patients attending a periodontal clinic during a period of 1 year. Samples from the gingival sulcus and the pharynx were collected for detection of *C. trachomatis*. Patients who attended the periodontal clinic were divided into two groups: those without periodontitis and those with periodontitis. For the purpose of performing other statistical analyses, all patients were also divided by gender and age.

**Results:**

From the total of 76 patients, in the group without periodontitis, 61% were positive for *C. trachomatis* in the gingival sulcus and 63.4% in the pharynx; in the periodontitis group, 45.7% were positive in the sulcus and 40% in the pharynx. When we compared patients by gender or age, no statistical difference was found.

**Conclusions:**

The prevalence of *C. trachomatis* in this group was 53.9% in the gingival sulcus and pharynx of the studied patients.

## 1 INTRODUCTION

Normal microbiota of the human oral cavity includes more than 500 different bacterial species including *Bacteroidetes*, *Actinobacteria*, *Proteobacteria*, *Euryarchaeota*, and others. These microorganisms have the ability to colonize gingival crevices and the outer surface of the tooth (Dewhirst et al., 2010; Reynolds‐Campbell, Nicholson, & Thoms‐Rodriguez, 2017). The oral microenvironment provides conditions for the establishment of microorganisms that are not usually considered normal oral microbiota and that are associated with periodontitis, poor hygiene, and/or a compromised immune system. These microorganisms may or not have a role in periodontal health or disease but the periodontal biofilm may be a source of dissemination and development of systemic infections by these pathogenic microorganisms (Vieira Colombo, Magalhães, Hartenbach, Martins do Souto, & Maciel da Silva‐Boghossian, 2016), including cardiovascular disease, adverse pregnancy outcomes, rheumatoid arthritis, inflammatory bowel disease and colorectal cancer, respiratory tract infections, and abscesses (Chan et al., 2016).

*Chlamydia trachomatis* is a Gram‐negative *obligate intracellular* bacterium with a propensity for the mucous membranes of their host. *C. trachomatis* is a common pathogen of the genital tract; however, it can live in any mucous membrane to which it adheres, including the mucosa of the oral cavity (Reed, Lopatin, Foxman, & Burt, 2000), pharynx, and other extra genital areas (Chan et al., 2016).

Although *C. trachomatis* extragenital infections are not considered risk factors because they are asymptomatic, it is important to know their prevalence and incidence because they serve as a reservoir and promote the propagation of antibiotic‐resistant strains (Chan et al., 2016; Danby et al., 2016).

For all these reasons, we focused on detecting the presence of *C. trachomatis* in the gingival sulcus and pharynx of patients with or without periodontal disease.

## 2 MATERIALS AND METHODS

This was a cross‐sectional observational study of patients who attended the periodontal unit of the School of Dentistry of the Universidad Autonoma de Nuevo Leon in Monterrey, Mexico. A total of 76 patients were randomly selected from 6,127 patients who attended the clinic during 1 year. Patients were divided into two groups: (a) periodontitis group, patients with active periodontitis, regardless of their stage of disease (mild, moderate, or severe) and (2) patients without periodontitis or gingivitis.

The inclusion criteria were as follows: men or women between 18 and 80 years of age at the time of the dental clinic visit; patients who had or have any of these diseases or symptoms such as conjunctivitis, xerostomia, otitis, rhinosinusitis, urinary infection, polyuria, urine sand, abortions, preeclampsia, or premature birth. The exclusion criteria were patients with a history of antibiotic treatment for *C. trachomatis* prior to the dental consultation, periodontal surgery in the past year, systemic diseases such as diabetes mellitus, Crohn disease, systemic lupus erythematosus, or ulcerative colitis. From the total of 76 patients, 35 were in the periodontitis group and 41 in the without periodontitis group. All patients signed a written informed consent and filled the data card. This study was approved by the Ethics Committee of the UANL School of Dentistry with registration number SPSI 010613‐00192.

### 2.1 Periodontal clinical evaluation to select the study groups

A single dental professional screened all patients. The indices obtained were periodontal pocket depth, defined as the distance between the gingival margin and the bottom of the periodontal pocket (Kim et al., 2017) which was evaluated with the periodontal probing clinical insertion level (Beltrán‐Aguilar, Eke, Thornton‐Evans, & Petersen, 2012); Turesky's modification of the “Quigley–Hein Plaque Index,” where plaque on the vestibular and lingual surfaces of the entire dentition is ranked on a scale of 0–5 after using a revealing agent. After all the surfaces are evaluated an average has to be calculated (Turesky, Gilmore, & Glickman, 1970); and the gingival bleeding index (Bessa Rebelo & de Queiroz, 2011).

### 2.2 Presence of *C. trachomatis* in the gingival sulcus and pharynx

Gingival sulcus samples were collected from each patient using sterile periodontal Gracey curettes (5/6); after that gingival curettage was done. Pharyngeal swabs were collected from each lateral posterior wall, including tonsillar crypts. Samples was transferred separately into a microtube with sterile saline solution and preserved at room temperature before extending on a slide and fixing with 90% alcohol.

**FIGURE 1:**
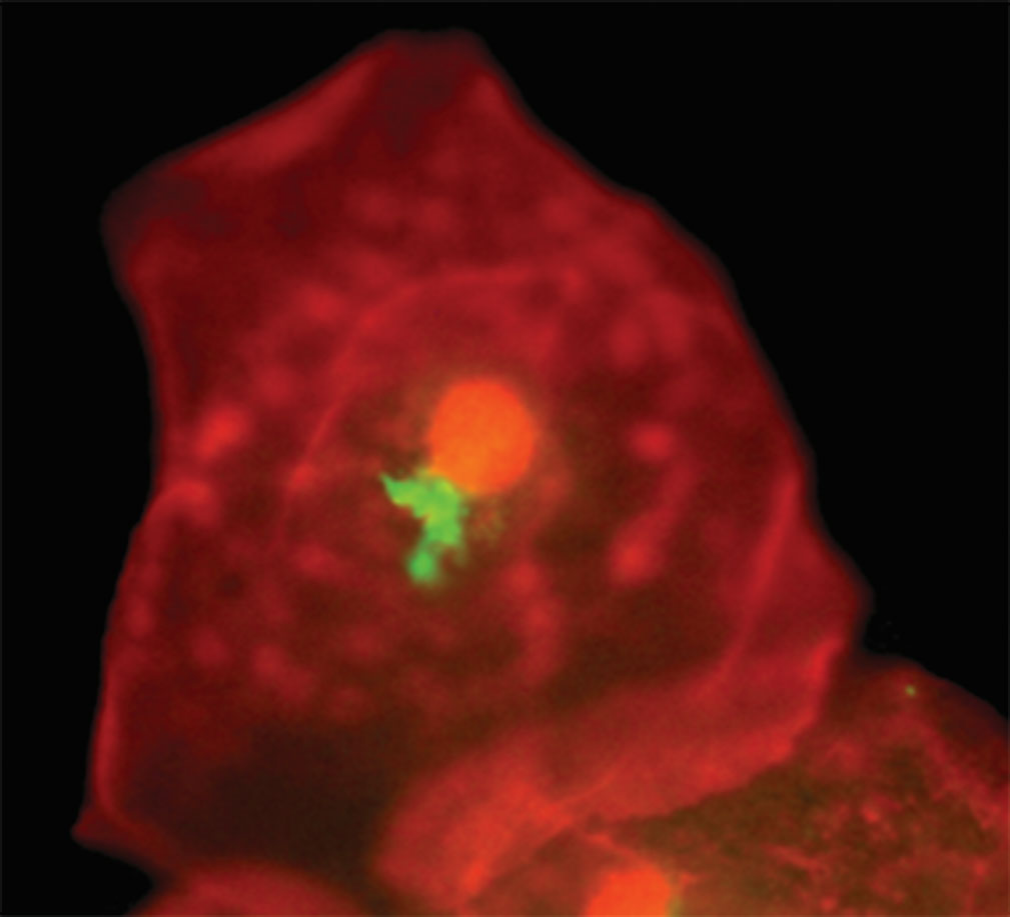
Representative image of a positive test for *Chlamydia trachomatis* in the gingival sulcus by fluorescence microscopy, 100×

*C. trachomatis* was detected in samples by direct immunofluorescence (DIF) using the Chlamydia DIF (bioMerieux®, Lyon, France) according to the instructions of the manufacturer. This method is based on the association of two fluorescein‐conjugated monoclonal antibodies directed each to different antigenic structures. The first monoclonal antibody recognizes an epitope located on the species antigen, the major outer membrane protein (MOMP); the second monoclonal antibody is directed against a genotype antigen and recognizes specific lipopolysaccharide structures. These two antibodies have complementary activity and allow the identification of the 15 serotypes of *C. trachomatis* in their different stages of evolution: elementals bodies, reticulated bodies, and inclusions. Samples were observed under a fluorescence microscope (Axiostar, Carl Zeiss). Visualization of more than 10 stained elementary bodies (green fluorescence) under fluorescence microscopy was accepted as a positive result (Figure 1).

### 2.3 Statistical analysis

Data analyses were conducted using SPSS statistical software version 22.0 (IBM Corp., Armonk, NY). *P* values were calculated using Fisher's exact or *X* ^2^.

## 3 RESULTS

### 3.1 Clinical data

A total of 76 patients were considered for this study, 34 men and 42 women with a mean age of 40.78 ± 14.87. Nineteen were in the 18–28 years age group, of which 12 (63.1%) were positive for *C. trachomatis* both in the gingival sulcus and the pharynx, respectively; 20 were 29–43 years old, of which 12 (60%) were positive in the gingival sulcus, and 11 (55%) in the pharynx; 25 were 44–58 years old, 12 (48%) were positive in the gingival sulcus and 12 (48%) in the pharynx; and 12 were 59–74 years old, 5 (41.6%) were positive in the gingival sulcus and 5 (41.6%) in the pharynx (Table 1).

**TABLE 1:** Patients from a periodontal clinic who are positive for *Chlamydia trachomatis* in the gingival sulcus or pharynx

	Presence of *C. trachomatis*	
Age range, years	Gingival sulcus, *n* (%)	Pharynx, *n* (%)
18–28	12/19 (63.1)	12/19 (63.1)
29–43	12/20 (60)	11/20 (55)
44–58	12/25 (48)	12/25 (48)
58–74	5/12 (41.6)	5/12 (41.6)

In order to elucidate if periodontic disease plays a role in the presence of *C. trachomatis*, patients were divided into two groups: without periodontitis (*n* = 41) and with periodontitis (*n* = 35). *C. trachomatis* was detected in the gingival sulcus of 25 (60.9%) patients from the without periodontitis group and 16 (45.7%) from the periodontitis group. No significant differences were found. In the case of pharynx samples, data were similar. A total of 26 (63.4%) patients without periodontitis and 14 (40%) cases with periodontitis were positive for *C. trachomatis* (Table 2).

**TABLE 2:** Positive cases of *Chlamydia trachomatis* in gingival sulcus or pharynx samples in periodontitis and without perdiodontitis groups

Sample	Preriodontitis *n* (%)		Without periodontitis, *n* (%)	
	Positive	Negative	Positive	Negative
Gingival sulcus	16 (45.7)	19 (54.3)	25 (60.9)	24 (39.1)
Pharynx	14 (40)	21 (60)	26 (63.4)	23 (36.6)

### 3.2 The presence of *C. trachomatis* in the gingival sulcus and pharynx

A total of 41 cases out of 76 (53.9%) were positive for *C. trachomatis* in the gingival sulcus. For comparison, we divided patients into males and females and into four age ranges. Of the gender group, 58% men and 50% women were positive for *C. trachomatis* in the gingival sulcus or pharynx samples (*p* < .05). Comparisons between gender and age in positive cases in the gingival sulcus and pharynx did not show a significant difference (Tables 3 and 4) but those between 18 and 28 years of age had a greater percentage of cases.

**TABLE 3:** Data of positive cases of *Chlamydia trachomatis* in the gingival sulcus in male or female groups and total cases comparing age

Age range, years	Male, *n* (%)	Female, *n* (%)	Total cases, *n* (%)
18–28	8/11 (72.7)	4/8 (50)	12/19 (63.1)
29–43	5/7 (71.4)	7/13 (53.8)	12/20 (60)
44–58	4/9 (44.4)	8/16 (50)	12/25 (48)
58–74	3/7 (42.8)	2/5 (40)	5/12 (41.6)

**TABLE 4:** Data of positive cases of *Chlamydia trachomatis* in the pharynx in male or female groups and total of cases comparing age

Age range, years	Male, *n* (%)	Female, *n* (%)	Total cases, *n* (%)
18–28	8/11 (72.7)	4/8 (50)	12/19 (63.1)
29–43	5/7 (71.4)	6/13 (46.1)	11/20 (60)
44–58	4/9 (44.4)	8/16 (50)	12/25 (48)
58–74	3/7 (42.8)	2/5 (40)	5/12 (41.6)

## 4 DISCUSSION

*C. trachomatis* is the most common sexually transmitted bacterial infection, affecting over 100 million people each year (Lanjouw et al., 2016) and 80% of infected women are asymptomatic (Rours et al., 2016). Detection of these bacteria in genital and extra genital tissues is important, in order to reduce morbidity and disease transmission (Danby et al., 2016; Dukers‐Muijrers, Schachter, van Liere, Wolffs, & Hoebe, 2015).

Studies in infertile couples from Northeastern Mexico, diagnosed with genital infection, have a high prevalence of chronic *C. trachomatis* infection (more than 90%) (Gallegos‐Avila et al., 2009). In women, uterine cervical duct obstruction by severe fibrosis, and endometritis were associated with the presence of *C. trachomatis*. Infertile men had sperm damage caused by oxygen‐free radicals produced by bacteria and leukocytes. Sperm phagocytosis, secondary to *C. trachomatis* infection, is also characteristic of seminal infection (Gallegos et al., 2008; Gallegos‐Avila, Ancer‐Rodríguez, Ortega‐Martínez, & Jaramillo‐Rangel, 2010). The high prevalence in this group may be due to the lack of symptoms in chronic infections, multiple sex partners, poor hygiene, and oral sex (Chan et al., 2016).

It is well known that oral sex is common in men and women and urogenital organisms are increasingly transmitted from the male urethra to the female pharynx and vice versa by fellatio. Bacteria reach the uterus during vaginal intercourse, suggesting that the presence of *C. trachomatis* in the pharynx, and others sites in the oral cavity reflect an important reservoir of infection (Danby et al., 2016).

The aim of this study was to detect the presence of *C. trachomatis* in the gingival sulcus and pharynx in dental patients and to determine if periodontal disease, gender, or age were determinants for their presence. Although it could be considered that the number of individuals evaluated for this study is low, the presence of *C. trachomatis* in the pharynx and gingival sulcus in 53% of these patients indicates that it is necessary to routinely search for these microorganisms in the community. Our results compared with other studies are contradictory, since others report a lower percentage of *C. trachomatis* in the sulcus and the pharynx (Chan et al., 2016; Danby et al., 2016; Dukers‐Muijrers et al., 2015; Reed et al., 2000). More information is necessary to identify these discrepancies. Nevertheless, as we mentioned before, when we take into account the high percentage found in infertile couples in this region of Mexico (≥90%) (Gallegos et al., 2008; Gallegos‐Avila et al., 2009), our figure of 53% of *C. trachomatis* in the pharynx and gingival sulcus is reliable and indicates the regional conditions of this high prevalence.

Recently, more attention has been given to the presence of *C. trachomatis* in extra genital areas, such as the pharynx, colon‐rectum, and others (Chan et al., 2016; Danby et al., 2016; Dukers‐Muijrers et al., 2015). Only one study has attempted to relate the presence of *C. trachomatis* with the development of periodontal disease. These authors detected *C. trachomatis* using a direct fluorescent monoclonal antibody (DFA). Only 7% (6/87) of the patients were positive and they concluded that *C. trachomatis* is not a periopathogenic agent. These data differ from ours, where we found that 40% of patients were positive for *C. trachomatis* in the periodontal sulcus (Reed et al., 2000).

In our patients, there was no statistical difference between the presence of *C. trachomatis* in the sulcus and the pharynx: 58% of men versus 50% (*p* > .05). These data are not in accordance with other similar research. Danby et al. (2016) reported positivity of *C. trachomatis* in 2.2% in men and in 1.7% women from pharyngeal swabs. In the same year, Chan et al. (2016) found a mean between women and men of 1.7 and 1.6%, respectively. More clinical information and an analysis of applied diagnosis techniques are necessary. We need more information in order to clear this discrepancy. Although we did not find a statistical difference between periodontitis and no periodontitis groups, ages or gender, it is important to note the presence of a genital microorganism in the oral cavity and to elucidate the population with a higher risk.

In the opinion of some authors, oral infections and poor oral health can provoke the introduction of oral microorganisms into the bloodstream or the lymphatic system. The subsequent attachment and multiplication of these bacteria on tissues or organs can lead to focal oral infections. Pathogenic agents may also remain at their primary oral site but released toxins can reach an organ or tissue via the bloodstream and cause metastatic infection that causes bacteremia, and heart, head, neck, respiratory system, and gastrointestinal system infections (Dukers‐Muijrers et al., 2015).

Among the limitations of this study is the use of DIF for the detection of *C. trachomatis*. Molecular techniques, such as polymerase chain reaction, are more commonly used for detection; however, DIF was chosen because of its rapid result and low cost since patients were attending the clinic for dental treatment.

Our group is considering the implementation of an extragenital test in the public health setting that may lead to an increase in case detection and optimal patient management of *C. trachomatis* infections and its sequela (Nall, Barr, McNeil, & Bachmann, 2016). Regional climate differences, migration of people, sexual education, and poor health care programs would be taken into account to consider *C. trachomatis* infection as a public health problem that requires major attention.

We conclude that the oral cavity it is an important reservoir for *C. trachomatis*. It is also important to identify its presence in order to improve clinical management to avoid reinfection in the community. A future study of a less diverse age group with a more thorough periodontal investigation and microbiological evaluation of the periodontal pockets to classify the severity of periodontal disease could be useful.

## CONFLICTS OF INTEREST

The authors declare that they have no conflicts of interest and that all have seen and approved the manuscript. We also declare that this manuscript is the authors' original work.
